# VSIG2 is associated with an immune-cold microenvironment and reduced response to PD-1 blockade in bladder cancer

**DOI:** 10.3389/fcimb.2026.1859986

**Published:** 2026-06-04

**Authors:** Jihao Wu, Pengyu Liang, Yunbo He, Mingxiao Zhang, Huanyi Lin, Guoping Li, Anze Yu

**Affiliations:** 1 Department of Urology, First Affiliated Hospital, Sun Yat-sen University, Guangzhou, Guangdong, China; 2 Department of Urology, Xiangya Hospital Central South University, Changsha, China; 3 Department of Urology, China-Japan Friendship Hospital, Beijing, China; 4 Department of Urology, Hainan General Hospital, (Hainan Affiliated Hospital of Hainan Medical University), Haikou, Hainan, China

**Keywords:** BLCA, immunotherapy, PD-1 blockade, tumor microenvironment, VSIG2

## Abstract

**Background:**

Bladder cancer is one of the most common malignancies worldwide, and only a subset of patients derives durable benefit from immune checkpoint blockade. An immune-cold tumor microenvironment, characterized by limited immune infiltration and impaired antitumor activity, is a major barrier to immunotherapy efficacy. However, the tumor-intrinsic factors that contribute to immune exclusion in bladder cancer remain incompletely understood. This study aimed to investigate the role of VSIG2 in shaping the immune microenvironment and immunotherapy response in bladder cancer.

**Methods:**

Integrative analyses were performed using bulk transcriptomic data, a Xiangya validation cohort, public single-cell RNA-seq datasets, and a Xiangya single-cell immunotherapy cohort. Immune-related transcriptional programs, immune cell infiltration, and cancer immunity cycle activity were evaluated by differential expression, enrichment, and immune deconvolution analyses. The cellular source and biological features of VSIG2 were further characterized at single-cell resolution. Clinical relevance was assessed by immunohistochemistry and response-associated analyses in immunotherapy-treated samples. Functional validation was performed in an MB49 syngeneic mouse model with VSIG2 knockdown combined with anti-PD-1 treatment.

**Results:**

VSIG2 was associated with an immune-cold phenotype in bladder cancer across multiple independent cohorts. In bulk transcriptomic analyses, VSIG2-high tumors exhibited reduced activity across several steps of the cancer immunity cycle, decreased infiltration of T cells, cytotoxic lymphocytes, and NK cells, and suppression of inflammatory, chemokine-related, and antigen-presentation programs. These findings were independently validated in the in-house cohort. Single-cell analyses showed that VSIG2 was predominantly enriched in malignant epithelial cells and marked tumor cell states characterized by weaker antigen-presentation, interferon-response, and immune interaction programs. In the in-house single-cell immunotherapy cohort, lower VSIG2 expression was associated with immunotherapy response and a more inflamed immune contexture. *In vivo*, VSIG2 silencing inhibited tumor growth, increased CD8-positive T-cell infiltration, and enhanced the antitumor efficacy of PD-1 blockade.

**Conclusions:**

VSIG2 is a tumor-associated molecule linked to immune exclusion and reduced responsiveness to PD-1 blockade in bladder cancer. Elevated VSIG2 expression marks malignant cell states with impaired immune engagement, whereas targeting VSIG2 enhances antitumor immunity and improves immunotherapy efficacy *in vivo*. These findings identify VSIG2 as a potential biomarker and therapeutic target for overcoming immunotherapy resistance in bladder cancer.

## Introduction

Bladder cancer (BC) remains one of the most common malignancies worldwide and continues to impose a substantial clinical burden because of its high rates of recurrence, progression, and therapeutic resistance (Comperat et al., 2022; Dyrskjot et al., 2023; Li et al., 2023; Yip et al., 2025). Although immune checkpoint blockade (ICB) has expanded treatment options for patients with advanced BC, durable responses are observed only in a limited subset of cases (Patel et al., 2020; Katoh et al., 2024; Lopez-Beltran et al., 2024). One major obstacle to successful immunotherapy is the presence of a non-inflamed or immune-excluded tumor microenvironment (TME), in which cytotoxic T lymphocytes fail to effectively infiltrate tumor nests or sustain antitumor activity (Mariathasan et al., 2018; Tran et al., 2021; Yu et al., 2023a). Elucidating the tumor-intrinsic factors that shape this immune context is therefore essential for improving the efficacy of immunotherapy in bladder cancer (Sweis et al., 2016; Spranger and Gajewski, 2018).

Emerging evidence suggests that tumor cell-intrinsic transcriptional and signaling programs actively regulate the immune composition of the TME (Yu et al., 2023b; Galassi et al., 2024; Roerden and Spranger, 2025). In addition to canonical immune checkpoint pathways, malignant cells can promote immune escape by limiting T-cell recruitment, attenuating inflammatory signaling, impairing antigen presentation, and fostering suppressive stromal or myeloid interactions. These processes ultimately contribute to the formation of an immune-cold tumor phenotype that is less responsive to ICB. Despite increasing recognition of these mechanisms, the molecular determinants that drive immune exclusion in bladder cancer remain incompletely understood.

V-set and immunoglobulin domain containing 2 (VSIG2) is a member of the immunoglobulin superfamily whose biological role in cancer remains poorly characterized (Zhou et al., 2022; Wang et al., 2025). Although aberrant VSIG2 expression has been reported in several tumor types, its relevance to tumor immunity and immunotherapy response has not been clearly defined (Yan et al., 2019; Xu et al., 2023; Zhu et al., 2023; Li et al., 2025; Ni et al., 2025). Given its structural features and potential involvement in intercellular communication, VSIG2 may participate in tumor-immune interactions and contribute to the establishment of an immunosuppressive microenvironment.

In the present study, we identified VSIG2 as a tumor-associated molecule linked to an immune-cold phenotype in bladder cancer. By integrating bulk transcriptomic analyses, public and institutional single-cell datasets, and *in vivo* functional validation, we found that elevated VSIG2 expression was associated with reduced immune infiltration, attenuated inflammatory and chemokine-related programs, and poor response to PD-1 blockade. At single-cell resolution, VSIG2 was predominantly enriched in malignant epithelial cells and marked tumor cell states associated with diminished immune interaction programs. Moreover, silencing VSIG2 enhanced CD8-positive T-cell infiltration and improved the antitumor efficacy of PD-1 blockade *in vivo*. Together, these findings identify VSIG2 as a key driver of immune exclusion and a potential therapeutic target for improving immunotherapy response in bladder cancer.

## Materials and methods

### Patient cohorts and clinical samples

Formalin-fixed, paraffin-embedded (FFPE) tumor specimens were retrospectively collected from patients with histologically confirmed bladder cancer who underwent surgical resection at the Xiangya Hospital of Central South University. Clinical information, including age, sex, tumor stage, grade, and follow-up data, was obtained from medical records. For a subset of patients receiving immune checkpoint blockade (ICB) therapy, treatment response and survival outcomes were recorded. All procedures involving human samples were approved by the Institutional Review Board, and written informed consent was obtained from all patients in accordance with the Declaration of Helsinki. In-house cohort and in-house scRNA cohort have been described previously (Hu et al., 2021; Yu et al., 2023b).

### Immunohistochemistry and quantification

FFPE tumor sections (4 μm) were deparaffinized, rehydrated, and subjected to antigen retrieval. Sections were incubated with primary antibodies against VSIG2, CD8 and PD-1, followed by appropriate HRP-conjugated secondary antibodies. Signals were visualized using DAB substrate and counterstained with hematoxylin.

VSIG2 expression was evaluated using a semi-quantitative scoring system based on staining intensity and the percentage of positive tumor cells. Patients were stratified into VSIG2-high and VSIG2-low groups according to the median VSIG2 expression level. CD8-positive and PD-1-positive immune cells were quantified by counting positive cells in multiple high-power fields and averaging across fields for each sample.

### Bulk transcriptomic and single-cell RNA-seq analyses

Publicly available bladder cancer bulk transcriptomic datasets and single-cell RNA-seq datasets, together with the in-house validation cohort (Hu et al., 2021; Yu et al., 2023b) and in-house scRNA-seq cohort (Hu et al., 2022), were analyzed to evaluate the association between VSIG2 expression and the tumor immune microenvironment. Tumors were stratified into VSIG2-high and VSIG2-low groups according to the median VSIG2 expression level unless otherwise specified. Differentially expressed genes between groups were identified and subjected to Gene Ontology (GO), Kyoto Encyclopedia of Genes and Genomes (KEGG), and Gene Set Enrichment Analysis (GSEA). Cancer immunity cycle activity was evaluated using GSVA-based signature scoring (Hanzelmann et al., 2013). Immune cell infiltration was estimated using MCPcounter (Becht et al., 2016) and other indicated immune deconvolution methods (Bindea et al., 2013; Li et al., 2020). For survival analysis, xCell-derived CD8-positive effector T-cell signatures were used to stratify patients in combination with VSIG2 expression (Aran et al., 2017).

For single-cell RNA-seq analysis, standard quality control, normalization, dimensionality reduction, clustering, and annotation procedures were performed (Hao et al., 2024). Malignant epithelial cells were re-clustered to identify distinct tumor cell states, and VSIG2-associated immune interaction programs were compared between VSIG2-high and VSIG2-low malignant epithelial cells. Cell-cell communication analysis was performed using CellChat v2 according to the developer’s standard workflow (Jin et al., 2025).

### Cell lines and culture

The murine bladder cancer cell line MB49 was obtained from Dr. Jian Huang from the Sun Yat-sen Memorial Hospital. Cells were cultured in DMEM or RPMI-1640 supplemented with 10% fetal bovine serum (FBS) and maintained at 37 °C in a humidified incubator with 5% CO_2_.

Cell lines were routinely tested for mycoplasma contamination and authenticated prior to use.

### Generation of VSIG2 knockdown models

To achieve stable silencing of VSIG2 in murine bladder cancer cells, short hairpin RNA (shRNA) sequences targeting mouse VSIG2 were designed and synthesized, while a non-targeting scrambled sequence was used as the negative control (shNC). These shRNA oligonucleotides were cloned into a lentiviral expression vector. Lentiviral particles were generated by co-transfecting the shRNA constructs with essential packaging plasmids into HEK293T cells. MB49 cells were subsequently transduced with the harvested lentiviral supernatant in the presence of polybrene and selected with puromycin (2 μg/mL) for at least 5 days to establish stable knockdown cell lines. The knockdown efficiency was rigorously validated at the protein levels through Western blot analysis, prior to all functional assays and *in vivo* experiments.

### Murine tumor models and treatment

All animal experiments were approved by the institutional animal care committee. Female C57BL/6 mice (6–8 weeks old) were subcutaneously injected with MB49 cells (control or VSIG2-modified). Tumor growth was monitored by caliper measurements and tumor volume was calculated using standard formulas. For immunotherapy experiments, mice were treated with anti–PD-1 antibodies or control IgG according to established protocols (Yu et al., 2025). At endpoint, tumors were harvested for downstream analysis of immune cell infiltration.

### Bioinformatics and statistical analysis

Publicly available datasets and/or in-house transcriptomic data were analyzed to evaluate the association between VSIG2 expression and immune infiltration. Immune cell abundance was estimated using established algorithms (Barbie et al., 2009; Newman et al., 2015) (e.g., CIBERSORT, ssGSEA). Survival analyses were performed using Kaplan–Meier methods and compared by log-rank tests. Correlations between VSIG2 expression and immune markers were assessed using Spearman or Pearson correlation coefficients. All statistical analyses were conducted using GraphPad Prism or R software. Data are presented as mean ± SEM unless otherwise specified. A two-sided *p* value < 0.05 was considered statistically significant.

## Results

### Integrative transcriptomic analyses identify VSIG2 as a marker of an immune-cold tumor phenotype in bladder cancer

**Figure 1:**
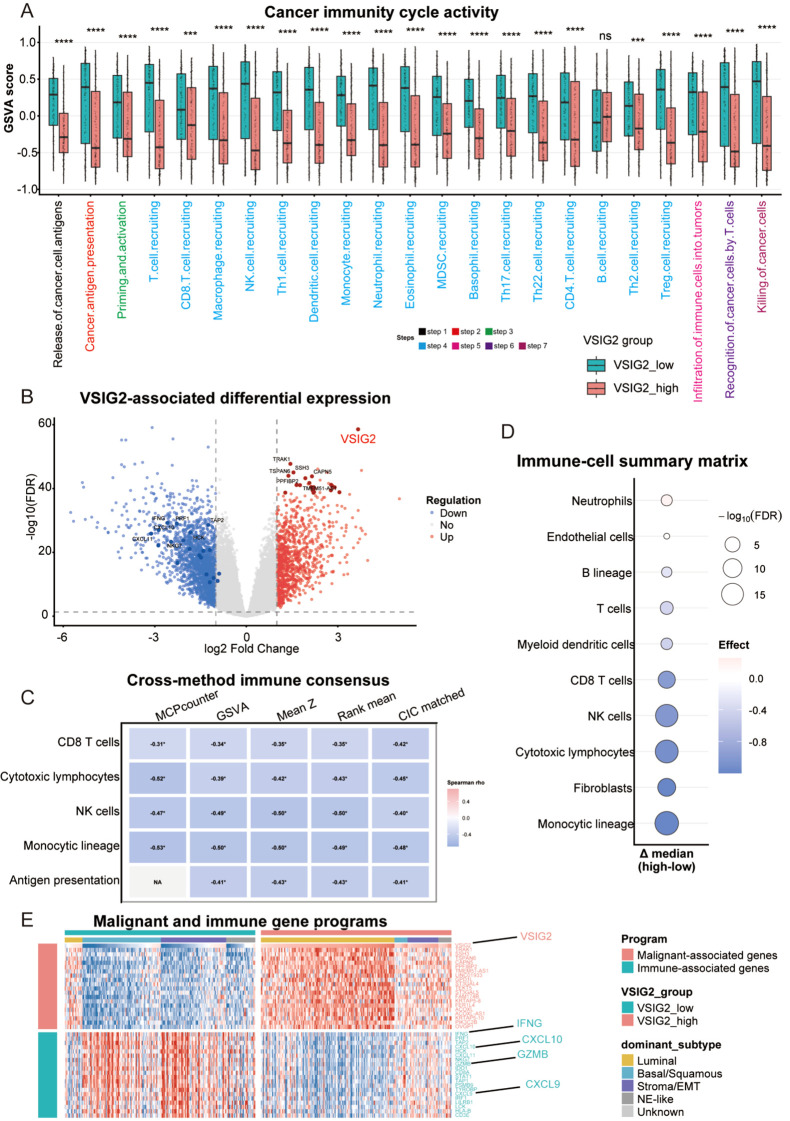
Integrative transcriptomic analyses identify VSIG2 as a candidate molecule associated with an immune-cold microenvironment in bladder cancer. **(A)** Comparison of cancer immunity cycle activity between the VSIG2-high and VSIG2-low groups based on GSVA scores. ***p<0.001, ****p<0.0001, ns indicates no significant difference. **(B)** Volcano plot showing differentially expressed genes between the VSIG2-high and VSIG2-low groups. **(C)** Cross-method immune consensus analysis integrating MCPcounter, GSVA, mean Z score, rank-based evaluation, and CIC-matched signatures. **(D)** Immune-cell summary matrix showing the relative abundance changes in stromal and immune cell populations between the two groups. Circle size indicates statistical significance, and color intensity indicates the magnitude of change. **(E)** Heatmap showing the expression patterns of malignant-associated and immune-associated gene programs in tumors stratified by VSIG2 expression.

To explore the potential role of VSIG2 in shaping the tumor immune microenvironment of bladder cancer, we first stratified tumors from The Cancer Genome Atlas (TCGA) cohort into VSIG2-high and VSIG2-low groups and performed transcriptomic analyses. Evaluation of the cancer immunity cycle revealed that elevated VSIG2 expression was associated with broad suppression of antitumor immune activity. Specifically, multiple key steps of the immunity cycle were significantly attenuated in the VSIG2-high group, including antigen presentation, T-cell priming and activation, and the recruitment of effector immune populations such as CD8^+^ T cells, NK cells, dendritic cells, and Th1 cells (**Figure 1A**). These findings suggested that VSIG2 upregulation was linked to impaired initiation and propagation of cancer immunity. We next compared the transcriptional profiles between the two groups. Differential expression analysis identified a distinct VSIG2-associated gene expression pattern, with VSIG2 itself among the most prominently upregulated genes in the VSIG2-high group (**Figure 1B**). In contrast, a substantial fraction of downregulated genes was related to immune activation and inflammatory responses, further indicating that VSIG2-high tumors may reside in a relatively immune-suppressed state. To more rigorously assess immune landscape differences, we integrated multiple immune deconvolution and signature-scoring approaches. Cross-method immune consensus analysis (Li et al., 2020) consistently demonstrated that signatures representing CD8^+^ T cells, cytotoxic lymphocytes, NK cells, monocytic lineage, and antigen presentation were all significantly reduced in VSIG2-high tumors across different algorithms (**Figure 1C**). Consistently, immune-cell summary matrix analysis (Bindea et al., 2013) confirmed a global reduction in immune infiltration in the VSIG2-high group, particularly affecting CD8^+^ T cells, NK cells, cytotoxic lymphocytes, monocytes, dendritic cells, T cells, and B-lineage cells (**Figure 1D**). Finally, gene program analysis (Hanzelmann et al., 2013) showed that tumors with high VSIG2 expression displayed enhanced malignant-cell-associated transcriptional features, accompanied by marked suppression of immune-associated effector molecules, including IFNG, CXCL10, GZMB, and CXCL9 (Rooney et al., 2015; Ayers et al., 2017) (**Figure 1E**). Together, these data identify VSIG2 as a tumor-associated molecule strongly linked to an immune-cold, weakly inflamed, and immune-excluded microenvironment in bladder cancer.

### Independent validation of the VSIG2-associated immune-cold phenotype in the in-house cohort

**Figure 2:**
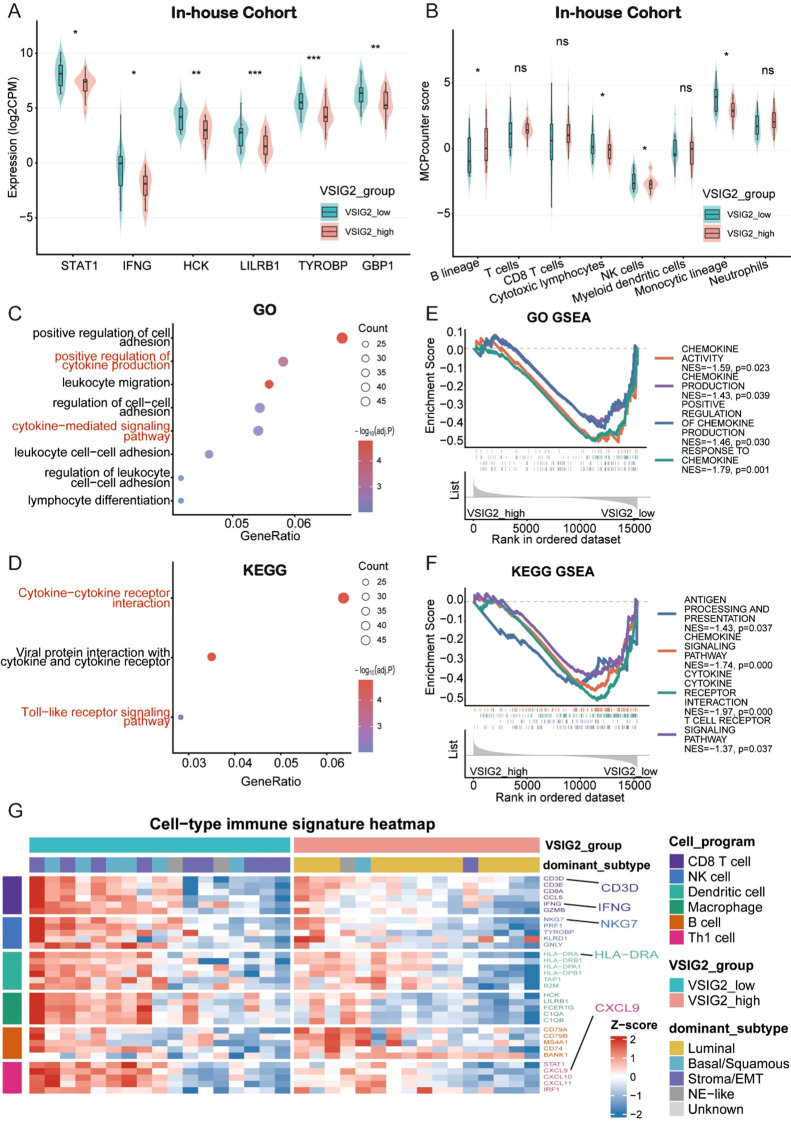
Validation of the association between VSIG2 and an immune-suppressed tumor microenvironment in the in-house cohort. **(A)** Violin plots showing the expression of representative immune-related genes in the in-house cohort stratified by VSIG2 expression. *p<0.05, **p<0.01, ***p<0.001. **(B)** MCPcounter analysis of the in-house cohort showing the relative abundance of major immune cell populations between the VSIG2-high and VSIG2-low groups. *p<0.05, ns indicates no significant difference. **(C)** GO enrichment analysis of differentially expressed genes associated with VSIG2, showing significant enrichment of immune-related biological processes. **(D)** KEGG pathway enrichment analysis of the VSIG2-associated genes. **(E, F)** GSEA plots showing that chemokine activity and cytokine-cytokine receptor interaction pathways were significantly suppressed in the VSIG2-high group. **(G)** Heatmap of cell-type immune signatures showing that tumors with high VSIG2 expression exhibited broadly reduced immune-related transcriptional programs across multiple immune cell populations, accompanied by lower expression of representative effector and antigen-presentation genes.

Consistent with the findings from the TCGA cohort, the in-house validation cohort further confirmed the association between elevated VSIG2 expression and an immune-cold tumor phenotype in bladder cancer. As shown in **Figure 2A**, VSIG2-high tumors exhibited significantly lower expression of multiple immune-related genes, including STAT1, IFNG, HCK, LILRB1, TYROBP, and GBP1, indicating attenuated immune activation in tumors with high VSIG2 expression. In parallel, MCPcounter analysis (Becht et al., 2016) demonstrated that VSIG2-high tumors harbored reduced infiltration of several major immune cell populations, including T cells, cytotoxic lymphocytes, NK cells, monocytic lineage cells, and neutrophils, whereas B-lineage cells showed no significant difference between the two groups (**Figure 2B**). To further characterize the biological processes associated with VSIG2, functional enrichment analysis of differentially expressed genes revealed significant enrichment of immune-related GO terms, including positive regulation of cytokine production, leukocyte migration, cytokine-mediated signaling, and regulation of leukocyte cell-cell adhesion (**Figure 2C**). KEGG pathway analysis similarly showed that VSIG2-associated transcriptional alterations were mainly enriched in cytokine-cytokine receptor interaction, viral protein interaction with cytokine and cytokine receptor, and Toll-like receptor signaling pathways (**Figure 2D**). Moreover, GSEA demonstrated that chemokine activity-related signatures were significantly suppressed in the VSIG2-high group (**Figure 2E**), while cytokine-cytokine receptor interaction and antigen processing and presentation pathways were also negatively enriched in these tumors (**Figure 2F**). In agreement with these results, the cell-type immune signature heatmap showed that VSIG2-high tumors displayed broadly decreased immune-related transcriptional programs across multiple immune cell compartments, together with lower expression of representative effector and antigen-presentation genes, including IFNG, NKG7, HLA-DRA, and CXCL9 (**Figure 2G**). Taken together, these findings independently validate the observations from **Figure 1** and further support that elevated VSIG2 expression is closely linked to diminished immune infiltration, impaired inflammatory signaling, and a non-inflamed tumor microenvironment in bladder cancer.

### Single-cell transcriptomic analysis localizes VSIG2 to malignant epithelial cell states associated with immune suppression

**Figure 3:**
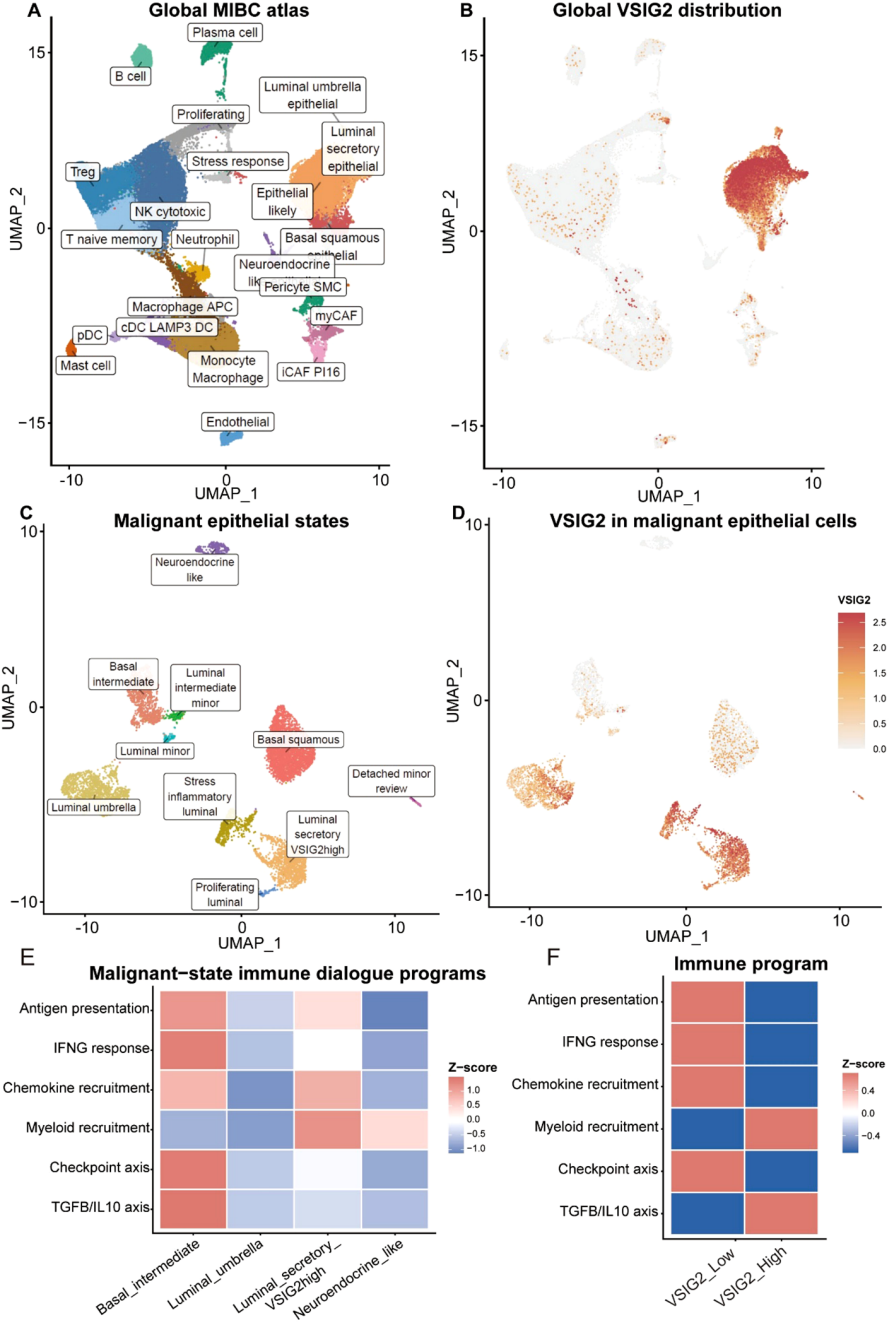
Single-cell transcriptomic analysis localizes VSIG2 to malignant epithelial cell states associated with immune suppression. **(A)** UMAP visualization of the global MIBC single-cell atlas showing the major cellular compartments. **(B)** Feature plot showing the global distribution of VSIG2 expression across the entire atlas. **(C)** UMAP visualization of malignant epithelial cell states. **(D)** Feature plot showing the distribution of VSIG2 expression within malignant epithelial cells. **(E)** Heatmap showing immune dialogue-related programs across distinct malignant epithelial states. **(F)** Comparison of immune programs between VSIG2-high and VSIG2-low malignant epithelial cells.

Building on the bulk transcriptomic findings from the TCGA and in-house cohorts, we next sought to define the cellular source of VSIG2 expression and its associated tumor cell states at single-cell resolution. As shown in **Figure 3A**, analysis of the global MIBC single-cell atlas (Lai et al., 2021; Tran et al., 2024; Liu et al., 2025) identified diverse malignant, immune, and stromal cell populations within the bladder tumor microenvironment. Mapping VSIG2 expression across the entire atlas revealed that VSIG2 was predominantly enriched in epithelial tumor cell compartments rather than in immune or stromal populations (**Figure 3B**), suggesting that VSIG2 is primarily a tumor cell-associated molecule in bladder cancer. To further characterize its distribution within malignant cells, we re-clustered the epithelial compartment and identified multiple transcriptionally distinct malignant epithelial states, including luminal umbrella, basal intermediate, luminal intermediate, stress inflammatory luminal, proliferating luminal, basal squamous, detached minor, neuroendocrine-like, and luminal secretory VSIG2-high populations (**Figure 3C**). Within these malignant epithelial subsets, VSIG2 expression was not uniformly distributed, but was preferentially enriched in specific epithelial states, particularly luminal-like subpopulations (**Figure 3D**). To further assess whether these VSIG2-enriched tumor states exhibited distinct immune interaction properties, we next examined malignant-state immune dialogue programs. As shown in **Figure 3E**, different malignant epithelial states displayed marked heterogeneity in antigen presentation, IFNG response, chemokine recruitment, myeloid recruitment, checkpoint axis, and TGFB/IL10 axis programs, with the luminal secretory VSIG2-high state showing relatively weaker antigen presentation, IFNG response, and chemokine recruitment signatures. Consistently, direct comparison between VSIG2-high and VSIG2-low malignant epithelial cells demonstrated that VSIG2-high tumor cells exhibited decreased antigen presentation, IFNG response, chemokine recruitment, and checkpoint-associated programs, whereas myeloid recruitment and TGFB/IL10-related signaling were relatively enriched in VSIG2-high cells (**Figure 3F**). Taken together, these findings indicate that VSIG2 is mainly expressed by malignant epithelial cells and marks a distinct tumor cell state associated with impaired immune activation and a more immunosuppressive interaction landscape in bladder cancer.

### Immune ecosystem remodeling associated with VSIG2-high malignant states

**Figure 4:**
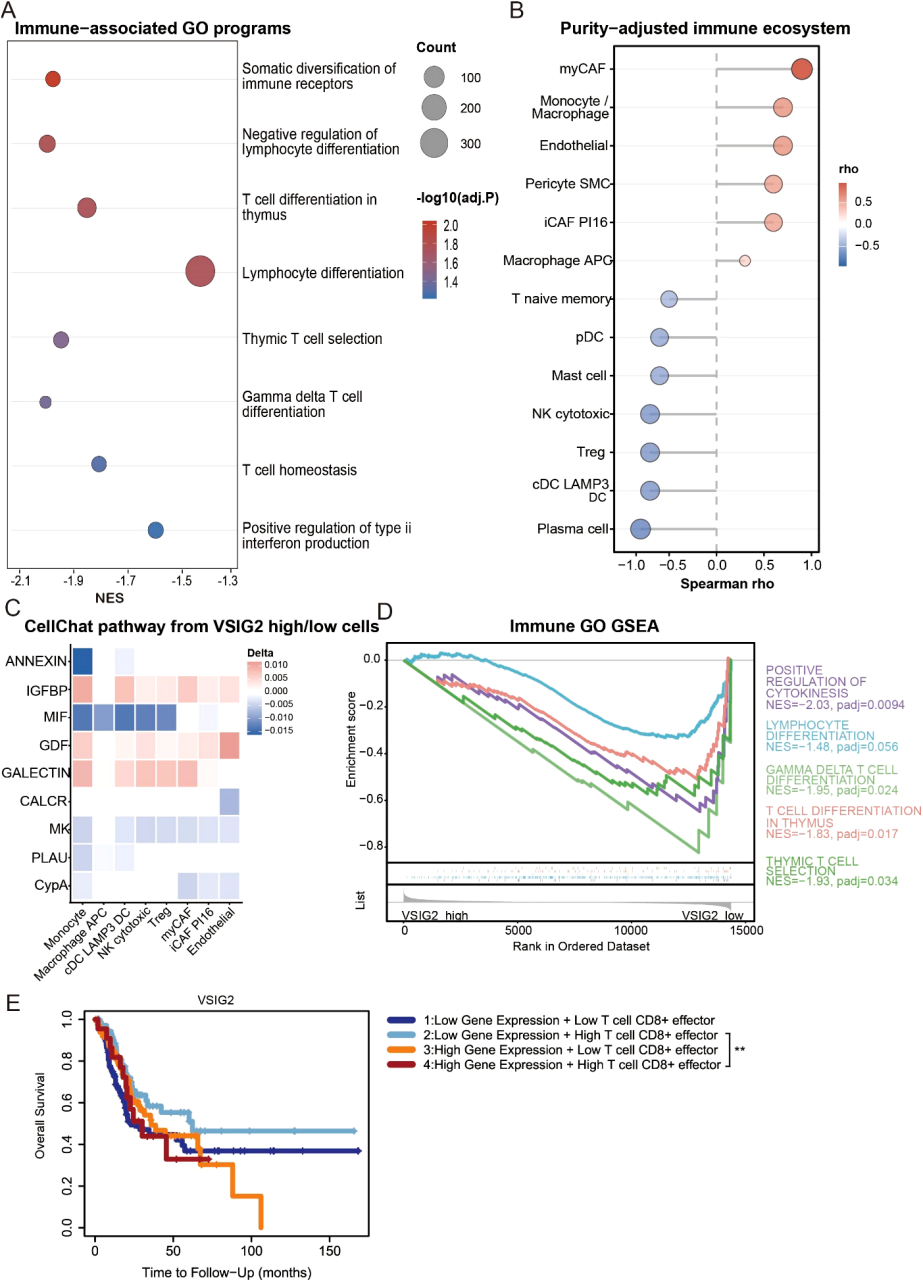
Immune ecosystem remodeling associated with VSIG2-high malignant states. **(A)** Immune-associated GO program analysis showing enrichment of VSIG2-related transcriptional alterations in biological processes. **(B)** Purity-adjusted immune ecosystem analysis showing correlations between VSIG2 expression and tumor microenvironment cell populations. **(C)** CellChat pathway analysis showing rewiring of intercellular communication pathways associated with VSIG2 status. **(D)** GSEA plots showing suppression of multiple immune-related biological processes in the VSIG2-high group. **(E)** Kaplan-Meier overall survival analysis stratified by VSIG2 expression and xCell-derived CD8^+^ effector T-cell infiltration. **p<0.01.

Having identified VSIG2 as a malignant epithelial cell-associated molecule at single-cell resolution, we next explored how VSIG2-high tumor states were linked to broader immune ecosystem remodeling. As shown in **Figures 4A**, immune-associated GO program analysis revealed that VSIG2-related transcriptional alterations were enriched in multiple immune regulatory processes, including somatic diversification of immune receptors, negative regulation of lymphocyte differentiation, T-cell differentiation in thymus, lymphocyte differentiation, thymic T-cell selection, γδ T-cell differentiation, T-cell homeostasis, and positive regulation of type II interferon production. To further characterize the immune context associated with VSIG2, purity-adjusted immune ecosystem analysis (Yoshihara et al., 2013) demonstrated that VSIG2 expression was positively correlated with stromal and myeloid-associated populations, including myCAF, monocyte/macrophage, endothelial cells, pericyte SMC, and iCAF PI16, whereas it was negatively correlated with multiple immune effector populations, including T naive memory cells, pDCs, mast cells, NK cytotoxic cells, Treg cells, cDC LAMP3 DCs, and plasma cells (**Figure 4B**). In parallel, CellChat pathway analysis (Jin et al., 2025) showed substantial rewiring of intercellular communication networks associated with VSIG2 status, particularly involving ANNEXIN, IGFBP, MIF, GDF, GALECTIN, CALCR, MK/MDK, PLAU, and CypA signaling pathways across myeloid, stromal, and lymphoid compartments (**Figure 4C**). Consistent with these observations, GSEA further demonstrated that multiple immune-related biological processes, including positive regulation of cytokinesis, lymphocyte differentiation, gamma-delta T-cell differentiation, T cell differentiation in thymus, and thymic T-cell selection, were significantly suppressed in the VSIG2-high group (**Figure 4D**). Finally, survival analysis based on the xCell-derived CD8+ effector T-cell signature (Aran et al., 2017) showed that patients with high VSIG2 expression combined with low CD8+ effector T-cell infiltration had the worst overall survival, whereas tumors with lower VSIG2 expression and higher CD8+ effector T-cell abundance were associated with more favorable outcomes (**Figure 4E**). Taken together, these findings indicate that VSIG2-high malignant states are associated with broad immune ecosystem remodeling characterized by reduced effector immune infiltration, altered stromal-myeloid interactions, and inferior clinical prognosis in bladder cancer.

### Single-cell validation in the in-house cohort links reduced VSIG2 expression to immunotherapy response

**Figure 5:**
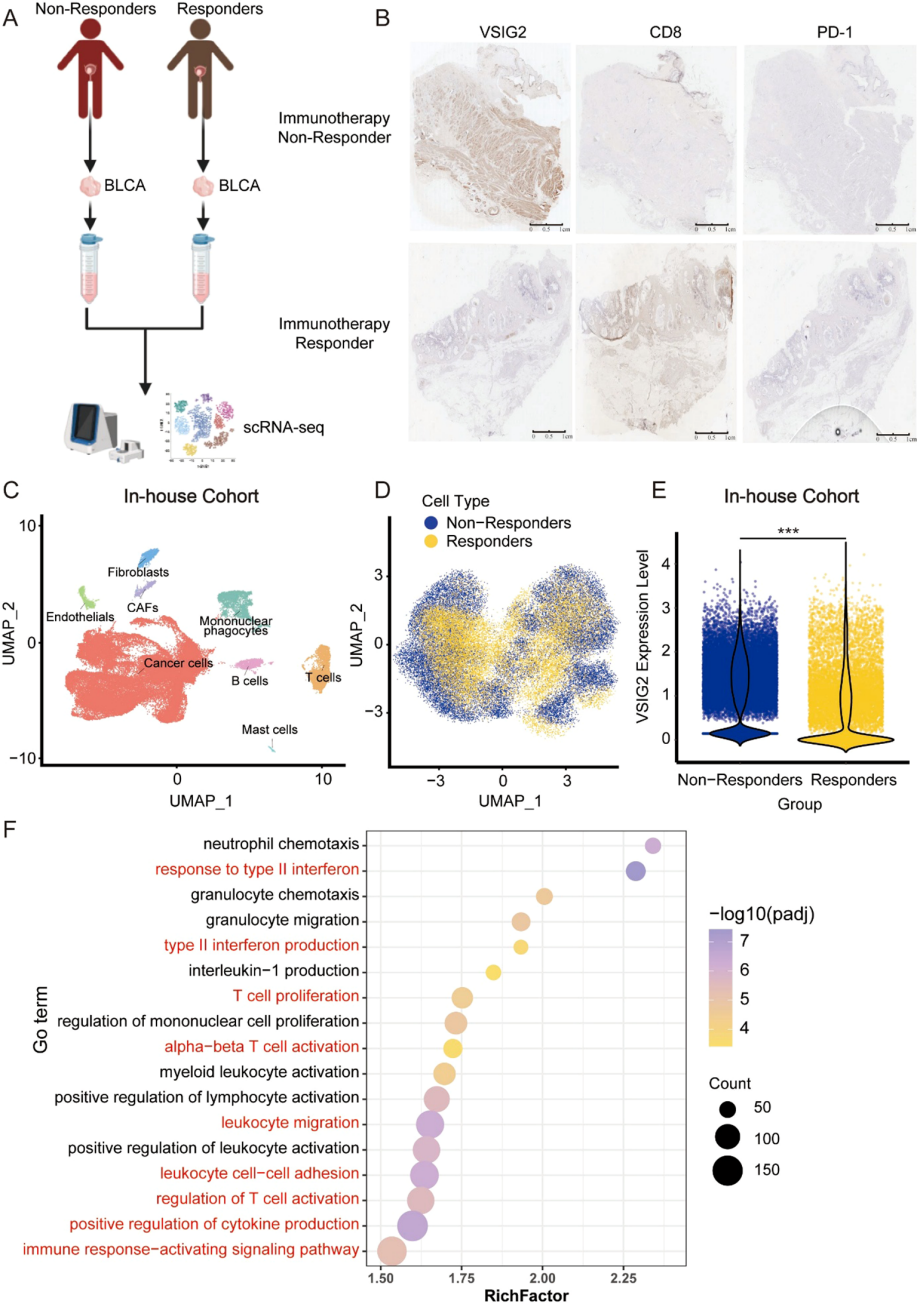
Single-cell validation in the in-house cohort links reduced VSIG2 expression to immunotherapy response. **(A)** Schematic overview of the in-house bladder cancer scRNA-seq cohort, including tumors from immunotherapy responders and non-responders. **(B)** Representative immunohistochemical staining of VSIG2, CD8, and PD-1 in tumors from immunotherapy non-responders and responders. **(C)** UMAP visualization of the in-house scRNA-seq cohort. **(D)** UMAP plot of the cancer cells colored by treatment response group. **(E)** Violin plot showing theVSIG2 expression the cancer cells from responder and non-responder samples. ***p<0.001. **(F)** GO enrichment analysis of responder-associated transcriptional programs.

To further validate the clinical relevance of VSIG2 at single-cell resolution in an independent immunotherapy-treated cohort, we performed scRNA-seq analysis on bladder cancer samples from immunotherapy responders and non-responders in the in-house cohort (**Figure 5A**). Immunohistochemical staining further showed that non-responder tumors exhibited higher VSIG2 expression but lower CD8 and PD-1 staining, whereas responder tumors displayed reduced VSIG2 expression accompanied by stronger CD8 and PD-1 signals, supporting an inverse association between VSIG2 expression and an inflamed tumor immune microenvironment (**Figure 5B**). Single-cell profiling identified multiple major cellular compartments in the in-house cohort, including cancer cells, T cells, B cells, mast cells, mononuclear phagocytes, CAFs, fibroblasts, and endothelial cells (**Figure 5C**). Mapping cells according to treatment response status showed distinct transcriptional distributions between responder- and non-responder-derived cells across the single-cell landscape (**Figure 5D**). Notably, VSIG2 expression was significantly lower in responders than in non-responders (**Figure 5E**), further supporting the association between elevated VSIG2 expression and immunotherapy resistance. Functional enrichment analysis of responder-associated transcriptional programs revealed significant enrichment of immune activation-related biological processes, including neutrophil and granulocyte chemotaxis, response to type II interferon, interleukin-1 production, T-cell proliferation and activation, and immune response-activating signaling pathways (**Figure 5F**). Taken together, these findings provide single-cell validation in an independent clinical cohort that lower VSIG2 expression is associated with a more inflamed immune contexture and improved response to immunotherapy in bladder cancer.

### *In vivo* targeting of VSIG2 enhances antitumor immunity and sensitizes bladder tumors to PD-1 blockade

**Figure 6:**
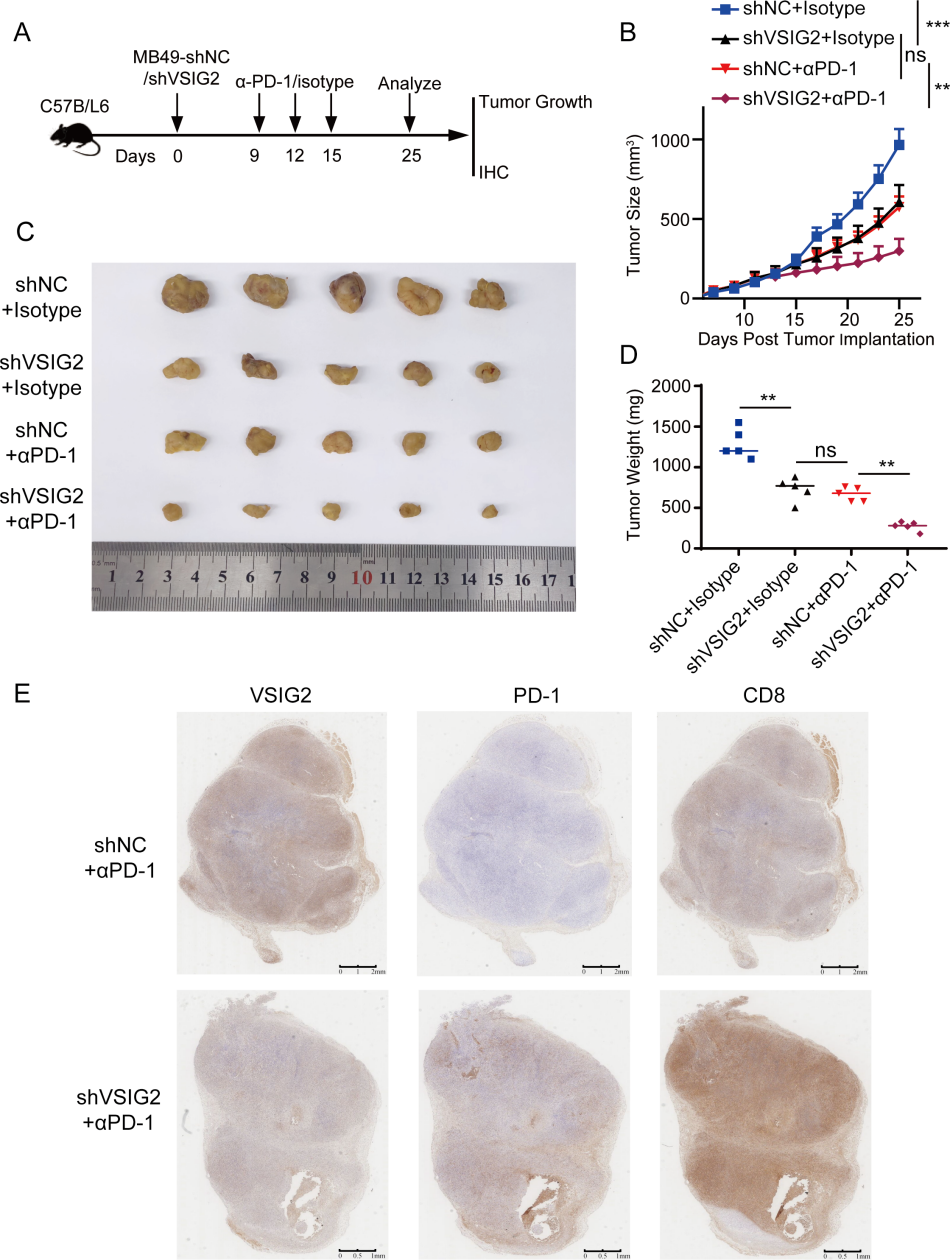
*In vivo* targeting of VSIG2 enhances antitumor immunity and sensitizes bladder tumors to PD-1 blockade. **(A)** Schematic illustration of the *in vivo* experimental design. **(B)** Tumor growth curves showing that VSIG2 knockdown significantly inhibited tumor progression and further enhanced the antitumor effect of PD-1 blockade *in vivo*. **p<0.01, ***p<0.001, ns indicates no significant difference. **(C)** Representative images of excised tumors from each treatment group **(D)** Tumor weight measurements confirming that combined VSIG2 silencing and PD-1 blockade produced the greatest reduction in tumor burden. **p<0.01, ns indicates no significant difference. **(E)** Representative immunohistochemical staining of VSIG2, PD-1, and CD8 in tumor tissues from the shNC plus anti-PD-1 and shVSIG2 plus anti-PD-1 groups.

Given the association between reduced VSIG2 expression and a more inflamed tumor microenvironment in clinical and single-cell analyses, we next investigated whether VSIG2 depletion could enhance the therapeutic efficacy of PD-1 blockade *in vivo*. As shown in **Figure 6A**, C57BL/6 mice were subcutaneously implanted with MB49 cells expressing either control shRNA or shVSIG2, followed by randomized to treat with anti-PD-1 antibody or isotype control according to the indicated schedule (Yu et al., 2025). Tumor growth analysis showed that both VSIG2 knockdown alone and anti-PD-1 treatment alone significantly suppressed tumor progression compared with the shNC plus isotype group, exhibiting comparable inhibitory effects (**Figure 6B**). Notably, the combination of VSIG2 knockdown and anti-PD-1 therapy resulted in the most pronounced suppression of tumor growth, indicating that loss of VSIG2 enhanced tumor sensitivity to PD-1 blockade. Consistently, excised tumors from the shVSIG2 plus anti-PD-1 group were visibly smaller than those from the other treatment groups (**Figure 6C**), and tumor weight measurements further confirmed that combined VSIG2 depletion and PD-1 blockade achieved the greatest antitumor effect *in vivo* (**Figure 6D**). Immunohistochemical analysis of tumor tissues demonstrated reduced VSIG2 staining in shVSIG2-treated tumors, accompanied by increased PD-1 and CD8 signals in the combination treatment group compared with the shNC plus anti-PD-1 group (**Figure 6E**), supporting enhanced immune activation and T-cell infiltration after VSIG2 silencing. Taken together, these findings demonstrate that VSIG2 depletion not only restrains bladder tumor growth but also potentiates the antitumor efficacy of PD-1 blockade, supporting a functional contribution of VSIG2 to immunotherapy resistance in bladder cancer.

## Discussion

In this study, we identified VSIG2 as a tumor cell-associated molecule closely linked to an immune-cold microenvironment and reduced immunotherapy responsiveness in bladder cancer (Chen and Mellman, 2017). Through integrative analyses of bulk transcriptomic datasets, public and institutional single-cell cohorts, and *in vivo* functional validation, we consistently found that elevated VSIG2 expression was associated with diminished immune infiltration, weakened inflammatory signaling, and inferior response to PD-1 blockade. In particular, VSIG2-high tumors exhibited reduced activity across multiple steps of the cancer immunity cycle, decreased expression of immune effector and antigen-presentation genes, and lower abundance of cytotoxic immune cell populations. These findings support a model in which VSIG2 marks a transcriptional and cellular tumor state associated with immune exclusion and immunotherapy resistance in bladder cancer.

One important finding of the present study is the robust association between VSIG2 expression and impaired antitumor immune infiltration. Across both TCGA and the in-house cohort, VSIG2-high tumors consistently displayed reduced immune-related gene expression and lower infiltration of multiple immune populations, especially T cells, cytotoxic lymphocytes, and NK cells. These results were further supported by immune signature analyses showing suppression of IFNG, CXCL9, CXCL10, NKG7, GZMB, and HLA-DRA-related programs in VSIG2-high tumors (Rooney et al., 2015; Ayers et al., 2017). Since effective immune checkpoint blockade depends on pre-existing immune activation and sufficient intratumoral T-cell infiltration, these findings suggest that elevated VSIG2 expression may define a non-inflamed tumor context that is intrinsically less permissive to productive antitumor immunity.

Our single-cell analyses provided additional insight into the cellular context of VSIG2 expression. In the public MIBC atlas, VSIG2 was predominantly localized to malignant epithelial cells rather than immune or stromal compartments, indicating that VSIG2 is mainly a tumor cell-associated factor in bladder cancer. Further stratification of malignant epithelial states showed that VSIG2 was enriched in specific luminal-like tumor cell populations characterized by weaker antigen-presentation, IFNG-response, and chemokine-recruitment programs. These observations are notable because they suggest that the immunological impact of VSIG2 may not simply reflect passive association with a suppressive microenvironment, but rather may arise from a tumor-intrinsic transcriptional state linked to reduced immune engagement. In parallel, the broader immune ecosystem associated with VSIG2-high tumors was characterized by relative depletion of effector immune populations and increased association with stromal and myeloid components, supporting the concept that VSIG2-high malignant states are embedded within a more suppressive tumor ecosystem (Binnewies et al., 2018; Kather et al., 2018; Mariathasan et al., 2018).

The in-house single-cell immunotherapy cohort further strengthened the clinical relevance of these observations. Compared with non-responders, responder tumors exhibited lower VSIG2 expression together with higher CD8 and PD-1 staining, and responder-associated transcriptional programs were enriched for interferon signaling, leukocyte migration, T-cell activation, and cytokine production. These findings are consistent with the view that low VSIG2 expression is linked to a more inflamed and immunologically active tumor state that is more compatible with benefit from checkpoint blockade. Although our data do not establish VSIG2 as a standalone predictive biomarker, they support its potential utility as a candidate indicator of immune context and immunotherapy sensitivity in bladder cancer.

Another important aspect of this study is the *in vivo* functional validation. In the MB49 model, VSIG2 silencing not only inhibited tumor growth but also enhanced the antitumor efficacy of PD-1 blockade, accompanied by increased CD8+ T-cell infiltration in tumor tissues. This result is important because it moves the study beyond correlative transcriptomic observations and supports a functional contribution of VSIG2 to immunotherapy resistance. From a translational perspective, these data suggest that targeting VSIG2 or the tumor programs associated with high VSIG2 expression may represent a strategy to improve sensitivity to immune checkpoint blockade, particularly in tumors with an immune-cold phenotype.

Despite these findings, several limitations should be acknowledged. First, although our results consistently associate VSIG2 with impaired immune activation and reduced response to immunotherapy, the precise molecular mechanisms by which VSIG2 shapes tumor-immune interactions remain unclear. In particular, whether VSIG2 directly regulates immune signaling, chemokine secretion, antigen presentation, or other pathways involved in T-cell recruitment and activation requires further investigation. Second, although we incorporated both public and institutional cohorts, the sample size of the single-cell immunotherapy cohort was still limited, and larger independent cohorts will be needed to confirm the robustness of these observations. Third, while our *in vivo* experiments support a functional role for VSIG2 in modulating antitumor immunity, our current *in vivo* validation is primarily limited to evaluating CD8+ T cell infiltration and PD-1 expression. Therefore, additional mechanistic studies and comprehensive validation in complementary models will be important to define its therapeutic relevance more precisely.

In conclusion, our study identifies VSIG2 as a tumor-associated marker linked to an immune-cold microenvironment and reduced immunotherapy response in bladder cancer. By integrating multi-cohort transcriptomic analyses, single-cell profiling, and *in vivo* validation, we show that elevated VSIG2 expression is associated with impaired immune infiltration, attenuated inflammatory programs, and resistance to PD-1 blockade. These findings provide a rationale for further investigation of VSIG2 as a candidate biomarker and therapeutic target for improving immunotherapy outcomes in bladder cancer.

## Data Availability

The datasets presented in this article are not readily available due to national regulatory guidelines concerning human sequencing data. Reasonable requests to access the datasets could be directed to Anze Yu at yuanz6@mail.sysu.edu.cn.

## References

[B1] Ayers M. Lunceford J. Nebozhyn M. Murphy E. Loboda A. Kaufman D. R. . (2017). xCell: digitally portraying the tissue cellular heterogeneity landscape. Genome Biol. 18, 220. doi: 10.1186/s13059-017-1349-1. PMID: 29141660 PMC5688663

[B2] Ayers M. . (2017). IFN-gamma-related mRNA profile predicts clinical response to PD-1 blockade. J. Clin. Invest. 127, 2930–2940. doi: 10.1172/JCI91190. PMID: 28650338 PMC5531419

[B3] Barbie D. A. Tamayo P. Boehm J. S. Kim S. Y. Moody S. E. Dunn I. F. . (2009). Systematic RNA interference reveals that oncogenic KRAS-driven cancers require TBK1. Nature 462, 108–112. doi: 10.1038/nature08460. PMID: 19847166 PMC2783335

[B4] Becht E. Giraldo N. A. Lacroix L. Buttard B. Elarouci N. Petitprez F. . (2016). Estimating the population abundance of tissue-infiltrating immune and stromal cell populations using gene expression. Genome Biol. 17, 218. doi: 10.1186/s13059-016-1070-5. PMID: 27765066 PMC5073889

[B5] Bindea G. Mlecnik B. Tosolini M. Kirilovsky A. Waldner M. Obenauf A. C. . (2013). Spatiotemporal dynamics of intratumoral immune cells reveal the immune landscape in human cancer. Immunity 39, 782–795. doi: 10.1016/j.immuni.2013.10.003. PMID: 24138885

[B6] Binnewies M. Roberts E. W. Kersten K. Chan V. Fearon D. F. Merad M. . (2018). Understanding the tumor immune microenvironment (TIME) for effective therapy. Nat. Med. 24, 541–550. doi: 10.1038/s41591-018-0014-x. PMID: 29686425 PMC5998822

[B7] Chen D. S. Mellman I. (2017). Elements of cancer immunity and the cancer-immune set point. Nature 541, 321–330. doi: 10.1038/nature21349. PMID: 28102259

[B8] Comperat E. Amin M. B. Cathomas R. Choudhury A. De Santis M. Kamat A. . (2022). Current best practice for bladder cancer: a narrative review of diagnostics and treatments. Lancet 400, 1712–1721. doi: 10.1016/S0140-6736(22)01188-6. PMID: 36174585

[B9] Dyrskjot L. Hansel D. E. Efstathiou J. A. Knowles M. A. Galsky M. D. Teoh J. . (2023). Bladder cancer. Nat. Rev. Dis. Primers 9, 58. doi: 10.1038/s41572-023-00468-9. PMID: 37884563 PMC11218610

[B10] Galassi C. Chan T. A. Vitale I. Galluzzi L. (2024). The hallmarks of cancer immune evasion. Cancer Cell. 42, 1825–1863. doi: 10.1016/j.ccell.2024.09.010. PMID: 39393356

[B11] Hanzelmann S. Castelo R. Guinney J. (2013). GSVA: gene set variation analysis for microarray and RNA-seq data. BMC Bioinf. 14, 7. doi: 10.1186/1471-2105-14-7. PMID: 23323831 PMC3618321

[B12] Hao Y. Stuart T. Kowalski M. H. Choudhary S. Hoffman P. Hartman A. . (2024). Dictionary learning for integrative, multimodal and scalable single-cell analysis. Nat. Biotechnol. 42, 293–304. doi: 10.1038/s41587-023-01767-y. PMID: 37231261 PMC10928517

[B13] Hu J. Othmane B. Yu A. Li H. Cai Z. Chen X. . (2021). 5mC regulator-mediated molecular subtypes depict the hallmarks of the tumor microenvironment and guide precision medicine in bladder cancer. BMC Med. 19, 289. doi: 10.1186/s12916-021-02163-6. PMID: 34836536 PMC8627095

[B14] Hu J. Chen J. Ou Z. Chen H. Liu Z. Chen M. . (2022). Neoadjuvant immunotherapy, chemotherapy, and combination therapy in muscle-invasive bladder cancer: a multi-center real-world retrospective study. Cell Rep. Med. 3, 100785. doi: 10.1016/j.xcrm.2022.100785. PMID: 36265483 PMC9729796

[B15] Jin S. Plikus M. V. Nie Q. (2025). CellChat for systematic analysis of cell-cell communication from single-cell transcriptomics. Nat. Protoc. 20, 180–219. doi: 10.1038/s41596-024-01045-4. PMID: 39289562

[B16] Kather J. N. Suarez-Carmona M. Charoentong P. Weis C. A. Hirsch D. Bankhead P. . (2018). Topography of cancer-associated immune cells in human solid tumors. Elife 7, e36967. doi: 10.7554/eLife.36967. PMID: 30179157 PMC6133554

[B17] Katoh M. Loriot Y. Brandi G. Tavolari S. Wainberg Z. A. Katoh M. (2024). FGFR-targeted therapeutics: clinical activity, mechanisms of resistance and new directions. Nat. Rev. Clin. Oncol. 21, 312–329. doi: 10.1038/s41571-024-00869-z. PMID: 38424198

[B18] Lai H. Cheng X. Liu Q. Luo W. Liu M. Zhang M. . (2021). Single-cell RNA sequencing reveals the epithelial cell heterogeneity and invasive subpopulation in human bladder cancer. Int. J. Cancer 149, 2099–2115. doi: 10.1002/ijc.33794. PMID: 34480339

[B19] Li T. Fu J. Zeng Z. Cohen D. Li J. Chen Q. . (2020). TIMER2.0 for analysis of tumor-infiltrating immune cells. Nucleic Acids Res. 48, W509–W514. doi: 10.1093/nar/gkaa407. PMID: 32442275 PMC7319575

[B20] Li F. Zheng Z. Chen W. Li D. Zhang H. Zhu Y. . (2023). Regulation of cisplatin resistance in bladder cancer by epigenetic mechanisms. Drug Resist. Update 68, 100938. doi: 10.1016/j.drup.2023.100938. PMID: 36774746

[B21] Li Y. Jiao P. Li D. Tian Y. Li H. Sun G. . (2025). Comprehensive analysis of aberrant m6A RNA modifications identifies prognostic biomarkers in non-small cell lung cancer. Int. J. Med. Sci. 22, 4396–4405. doi: 10.7150/ijms.119651. PMID: 41209572 PMC12595339

[B22] Liu Z. Mao X. Xie Y. Yan Y. Wang X. Mi J. . (2025). Single-cell RNA sequencing reveals a fibroblast gene signature that promotes T-cell infiltration in muscle-invasive bladder cancer. Commun. Biol. 8, 696. doi: 10.1038/s42003-025-08094-9. PMID: 40319103 PMC12049545

[B23] Lopez-Beltran A. Cookson M. S. Guercio B. J. Cheng L. (2024). Advances in diagnosis and treatment of bladder cancer. BMJ 384, e076743. doi: 10.1136/bmj-2023-076743. PMID: 38346808

[B24] Mariathasan S. Turley S. J. Nickles D. Castiglioni A. Yuen K. Wang Y. . (2018). TGFbeta attenuates tumour response to PD-L1 blockade by contributing to exclusion of T cells. Nature 554, 544–548. doi: 10.1038/nature25501. PMID: 29443960 PMC6028240

[B25] Newman A. M. Liu C. L. Green M. R. Gentles A. J. Feng W. Xu Y. . (2015). Robust enumeration of cell subsets from tissue expression profiles. Nat. Methods 12, 453–457. doi: 10.1038/nmeth.3337. PMID: 25822800 PMC4739640

[B26] Ni Q. Wang Y. Bian X. Qu Q. Shen B. Niu Y. . (2025). VSIG2 hinders gastric cancer progression by suppressing ANXA2-mediated NF-kappaB pathway activation. Acta Biochim. Biophys. Sin. (Shanghai) 57, 1834–1846. doi: 10.3724/abbs.2025202. PMID: 41185558 PMC12666673

[B27] Patel V. G. Oh W. K. Galsky M. D. (2020). Treatment of muscle-invasive and advanced bladder cancer in 2020. CA Cancer J. Clin. 70, 404–423. doi: 10.3322/caac.21631. PMID: 32767764

[B28] Roerden M. Spranger S. (2025). Cancer immune evasion, immunoediting and intratumour heterogeneity. Nat. Rev. Immunol. 25, 353–369. doi: 10.1038/s41577-024-01111-8. PMID: 39748116

[B29] Rooney M. S. Shukla S. A. Wu C. J. Getz G. Hacohen N. (2015). Molecular and genetic properties of tumors associated with local immune cytolytic activity. Cell. 160, 48–61. doi: 10.1016/j.cell.2014.12.033. PMID: 25594174 PMC4856474

[B30] Spranger S. Gajewski T. F. (2018). Impact of oncogenic pathways on evasion of antitumour immune responses. Nat. Rev. Cancer 18, 139–147. doi: 10.1038/nrc.2017.117. PMID: 29326431 PMC6685071

[B31] Sweis R. F. Spranger S. Bao R. Paner G. P. Stadler W. M. Steinberg G. . (2016). Molecular drivers of the non-T-cell-inflamed tumor microenvironment in urothelial bladder cancer. Cancer Immunol. Res. 4, 563–568. doi: 10.1158/2326-6066.CIR-15-0274. PMID: 27197067 PMC4943758

[B32] Tran M. A. Youssef D. Shroff S. Chowhan D. Beaumont K. G. Sebra R. . (2024). Urine scRNAseq reveals new insights into the bladder tumor immune microenvironment. J. Exp. Med. 221 (8), e20240045. doi: 10.1084/jem.20240045. PMID: 38847806 PMC11157455

[B33] Tran L. Xiao J. F. Agarwal N. Duex J. E. Theodorescu D. (2021). Advances in bladder cancer biology and therapy. Nat. Rev. Cancer 21, 104–121. doi: 10.1038/s41568-020-00313-1. PMID: 33268841 PMC10112195

[B34] Wang X. Hu R. Chen K. He K. Li Y. Gao J. . (2025). VSIG2 as a novel immunosuppressive ligand interacts with Nectin-2 to regulate T cell responses. J. Neuroinflamm. 23, 11. doi: 10.1186/s12974-025-03645-7. PMID: 41350674 PMC12797591

[B35] Xu J. Quan G. Huang W. Jiang J. (2023). VSIG2 promotes Malignant progression of pancreatic ductal adenocarcinoma by enhancing LAMTOR2-mediated mTOR activation. Cell. Commun. Signal. 21, 223. doi: 10.1186/s12964-023-01209-x. PMID: 37626304 PMC10463957

[B36] Yan H. Qu J. Cao W. Liu Y. Zheng G. Zhang E. . (2019). Identification of prognostic genes in the acute myeloid leukemia immune microenvironment based on TCGA data analysis. Cancer Immunol. Immunother. 68, 1971–1978. doi: 10.1007/s00262-019-02408-7. PMID: 31650199 PMC11028253

[B37] Yip W. Jaime-Casas S. Kothari A. Sullivan M. Ballas L. K. Escobar D. . (2025). Urothelial carcinoma: Perioperative considerations from top to bottom. CA Cancer J. Clin. 75, 528–551. doi: 10.3322/caac.70019. PMID: 40478748 PMC12593283

[B38] Yoshihara K. Shahmoradgoli M. Martinez E. Vegesna R. Kim H. Torres-Garcia W. . (2013). Inferring tumour purity and stromal and immune cell admixture from expression data. Nat. Commun. 4, 2612. doi: 10.1038/ncomms3612. PMID: 24113773 PMC3826632

[B39] Yu A. Fu J. Yin Z. Yan H. Xiao X. Zou D. . (2023a). Continuous expression of interferon regulatory factor 4 sustains CD8(+) T cell immunity against tumor. Res. (Wash D. C) 6, 271. doi: 10.34133/research.0271. PMID: 38178902 PMC10765897

[B40] Yu A. Hu J. Fu L. Huang G. Deng D. Zhang M. . (2023b). Bladder cancer intrinsic LRFN2 drives anticancer immunotherapy resistance by attenuating CD8(+) T cell infiltration and functional transition. J. ImmunoTher. Cancer 11 (10), e007230. doi: 10.1136/jitc-2023-007230. PMID: 37802603 PMC10565151

[B41] Yu A. Fu L. Jing L. Wang Y. Ma Z. Zhou X. . (2025). Methionine-driven YTHDF1 expression facilitates bladder cancer progression by attenuating RIG-I-modulated immune responses and enhancing the eIF5B-PD-L1 axis. Cell Death Differ. 32, 776–791. doi: 10.1038/s41418-024-01434-y. PMID: 39672819 PMC11982326

[B42] Zhou X. Khan S. Huang D. Li L. (2022). V-Set and immunoglobulin domain containing (VSIG) proteins as emerging immune checkpoint targets for cancer immunotherapy. Front. Immunol. 13, 938470. doi: 10.3389/fimmu.2022.938470. PMID: 36189222 PMC9520664

[B43] Zhu H. Li Y. Guo J. Feng S. Ge H. Gu C. . (2023). Integrated proteomic and phosphoproteomic analysis for characterization of colorectal cancer. J. Proteomics 274, 104808. doi: 10.1016/j.jprot.2022.104808. PMID: 36596410

